# Clinical characteristics and outcomes in critical patients with hemorrhagic fever with renal syndrome

**DOI:** 10.1186/1471-2334-14-191

**Published:** 2014-04-08

**Authors:** Hong Du, Ping-Zhong Wang, Jing Li, Lu Bai, Huan Li, Hai-Tao Yu, Wei Jiang, Ye Zhang, Jun-Ning Wang, Xue-Fan Bai

**Affiliations:** 1 Center for Infectious Diseases, Tangdu Hospital, Fourth Military Medical University, 569 Xinsi Rd, Baqiao District, Xi’an, Shaanxi 710038, China; 2 Center for Clinical Laboratory, Xijing Hospital, Fourth Military Medical University, Xi’an, Shaanxi, China; 3 Center for Clinical Laboratory, Tangdu Hospital, Fourth Military Medical University, Xi’an, Shaanxi, China

**Keywords:** Hemorrhagic fever with renal syndrome, Prognosis, Hantavirus

## Abstract

**Background:**

Hemorrhagic fever with renal syndrome (HFRS) has become an important public health concern because of the high incidence and mortality rates, and limited treatment and vaccination. Until now, clinical studies on characteristics and outcomes in critical patients with HFRS have been limited. The aim of this study was to observe the clinical characteristics and cumulative proportions surviving and explore the predictive effects and risk factors for prognosis.

**Methods:**

A detailed retrospective analysis of clinical records for critical HFRS patients was conducted. The patients enrolled were treated in the centre for infectious diseases, Tangdu Hospital, between January 2008 and August 2012. The clinical characteristics between the survivors and non-survivors were compared by Student’s t-test or Chi-square test. The risk clinical factors for prognosis were explored by logistic regression analysis. The predictive effects of prognosis in clinical and laboratory parameters were analyzed by receiver operating characteristic (ROC) curves. The cumulative proportions surviving at certain intervals in the critical patients were observed by Kaplan-Meier survival analysis.

**Results:**

Of the 75 patients enrolled, the cumulative proportion surviving was 70.7% at the second week interval, with a 28-day mortality rate of 36.3%. The non-survivors tended to have higher frequencies of agitation, dyspnea, conjunctival hemorrhage, coma, cardiac failure, acute respiratory distress syndrome (ARDS) and encephalopathy (P < .05). ARDS, conjunctival hemorrhage and coma were risk factors for death in the critical patients with HFRS. The non-survivors were found to have lower serum creatinine (Scr) levels (P < .001) and higher incidences of prolonged prothrombin time (PT) (P = .006), activated partial thromboplastin time (APTT) (P = .003) and elevated white blood cells (WBC) levels (P = .005), and the laboratory parameters mentioned above reached statistical significance for predicting prognosis (P < .05).

**Conclusion:**

The high fatality in critical patients with HFRS underscores the importance of clinicians’ alertness to the occurrence of potentially fatal complications and changes in biochemical status to ensure that timely and systematically supportive treatment can be initiated when necessary.

## Background

Hemorrhagic fever with renal syndrome (HFRS) is a rodent-borne disease caused by Hantavirus [1,2]. Hantaviruses are carried by rodents, and transmission to humans is caused by the inhalation of infected rodent excreta [3]. China is the most severe endemic area of HFRS in the world, with 30,000-50,000 cases reported annually, which account for > 90% of total numbers worldwide [4,5]. Shaanxi province is one of the most severely affected provinces in China. Xi’an is the center district of Shaanxi province and has had an increased incidence and mortality rate in the last three years [6]. Until now, it has been reported that all HFRS cases in this district are caused by hantaan virus (HTNV), a major serotype of Hantavirus [6], and the disease usually occurs in the spring and autumn/winter, with two incidence peaks [7]. Furthermore, a large number of patients in this epidemic area were adults, which was in sharp contrast to the predominantly pediatric cases seen in the dengue epidemics in southeast Asia [8,9].

The clinical course of HFRS is primarily characterized by fever, circulatory collapse with hypotension, hemorrhage and acute kidney injury (AKI) [2,10]. The typical disease progresses through five phases: febrile, hypotensive, oliguric, diuretic, and convalescent [11,12]. A hallmark of HFRS is capillary leak syndrome, which causes edema and hemorrhage, suggesting that the vascular endothelium is the primary target of virus infection [13,14]. In view of the rapidly expanding population and high mortality rate of infection in this district [6], it is essential to obtain a more comprehensive and better understanding of the clinical characteristics and outcomes in critical patients with this disease and to help clinicians engage in timely monitoring and effective supportive therapy in the early stages of the disease to improve the final survival rate.

## Methods

### Study participants

The medical charts of 356 typical HFRS patients who were treated in the Center for Infectious Diseases, Tangdu Hospital, between January 2008 and August 2012 were selected randomly and reviewed. The diagnosis of HFRS was made based upon the detection of specific IgM and IgG antibodies to HTNV in acute phase serum specimens by enzyme-linked immunosorbent assay (ELISA).

Based upon clinical classification of HFRS [15], the patients were classified into four types: (1) mild, defined as patients who had kidney injury without oliguria and hypotension; (2) moderate, defined as patients who had uremia, effusion (bulbar conjunctiva), hypotension, hemorrhage (skin and mucous membranes), and AKI with typical oliguria; (3) severe, defined as patients who had severe uremia, effusion (bulbar conjunctiva and either peritoneum or pleura), hemorrhage (skin and mucous membranes), hypotension and AKI with oliguria (urine output of 50–500 mL/day) for ≤ 5 days or anuria (urine output of < 100 mL/day) for ≤ 2 days; (4) critical, defined as patients who usually had one or more of the following complications compared with the severe patients: refractory shock (≥ 2 days), visceral hemorrhage, heart failure, pulmonary edema, brain edema, severe secondary infection, and severe AKI with oliguria (urine output of 50–500 mL/day) for > 5 days or anuria (urine output of < 100 mL/day) for > 2 days. Commonly, the so-called acute stage of the disease is defined as the period of febrile, hypotensive and oliguric phases. Overall, 75 cases were classified as critical type and were enrolled in this study. Furthermore, the outcome was defined as death or survival during the interval of being in hospital and after discharge with following up.

### Laboratory and imaging studies

Laboratory and imaging results that documented the clinical presentations and outcomes of the critical patients with HFRS complicated by ARDS were analyzed and compared. Biochemical tests of blood samples were performed using an autoanalyzer (Sysmex, XT-4000i, Japan; Hitachi, 7600–100, Japan), including basic metabolic, liver and renal function and glucose tests. Blood clotting functions were tested using hematology analyzers (CA7000, Sysmex, Japan; ACL, TOP700, United States). Chest and abdomen organs were visualized using X-ray radiography (PLOYMOBIL 2.5, Siemens, Germany) and ultrasonography (DC-6, MINDRAY, China). Computed tomography (CT) (CTTM64, Siemens, Germany) was performed in some patients. Cardiac function was measured using Cardiofax (1350p, NIHON KOHDEN, Japan) and ultrasonography (DC-7, MINDRAY, China). Arterial blood gases were measured using an automatic blood gas system (ABL80, Denmark). Hemo cultures were tested using an autoanalyzer (BD9120, United States; BD9050, United States).

### Definition of HFRS-related complications

Acute renal failure (ARF) was defined as the third stage of AKI according to the criteria of Acute Kidney Injury Network (AKIN) [16]. Patients who had manifestations of altered mental status (drowsiness, spasm, lethargy, agitation, or coma) were defined as having encephalopathy. Patients who had manifestations of acute respiratory distress including dyspnea, short breath, cyanosis accompanied with oxygenation index (PaO_2_/FiO_2_) ≤ 200 mmHg, were defined as exhibiting ARDS [17]. Gastrointestinal hemorrhage was defined as hematemesis or dark stools with hemodynamic instability and a rapid decrease in hemoglobin level to ≤ 7.0 g/dL. Secondary hyperglycemia was defined as an increase of blood-fasting sugar ≥ 7.1 mmol/L without primary diabetes. Pneumonia was clinically diagnosed according to the manifestations of cough, expectoration and chest distress with confirmation by X-ray radiography or/and chest CT. Concurrent bacteremia was defined as a positive bacterial growth from blood that was sampled for culture within 72 h after the patient was hospitalized. Secondary hypertension was defined as systolic blood pressure (SBP) ≥ 140 mmHg during the clinical course without primary hypertension. Cardiac failure was defined as hypotension and tachycardia based upon the fluid expansion with the confirmation by cardiac ultrasonography and invasive hemodynamic monitoring. Arrhythmia, including atrial premature beats, ventricular premature contraction, atrial fibrillation, ventricular fibrillation, supraventricular tachycardia, ventricular tachycardia and atrial ventricular block, was confirmed by electrocardiography (ECG).

### Statistical analysis

Statistical analysis was performed using SPSS 17.0 software (SPSS Inc., Chicago, IL, USA). Tables were created using Excel 2003 (Microsoft), and figures were created using GraphPad Prism 5 (GraphPad Software, San Diego CA). Continuous variables are presented as the mean ± SD and were analyzed by Kolmogorov-Smirnov’s test for normality of the distribution and Levene’s test for the homogeneity of variance. The variables were compared with Student’s t-test for normally distributed variables. For non-normally distributed variables, the nonparametric Mann–Whitney U-test was used. The frequencies and percentages are given for qualitative variables. Significant differences were tested by the chi-square test, and Fisher’s exact test was used when numbers were too small to perform the chi-square test. Spearman’s correlation coefficient was used to determine the relationship between the clinical manifestations, laboratory values, complications and survival outcome, respectively. Binary logistic regression analysis was used to identify clinical risk factors for prognosis. Predicting values for prognosis of the laboratory parameters were tested with receiver operating characteristic (ROC) curves and quantified by calculating the area under the curve (AUC) and the 95% confidence interval (CI). Kaplan-Meier analysis was used for the cumulative proportion surviving and 28-day mortality rate in the critical patients. A two-tailed P < .05 was considered statistically significant.

### Ethics statement

This retrospective study was reviewed and approved by the Institutional Review Board of Tangdu Hosipital, and the patients’ medical records were anonymized and de-identified prior to analysis.

## Results

### Clinical, demographic and epidemiologic conditions of the critical patients

Of the 75 critical patients, 46 survived and 29 died; 59 patients (78.7%) were male, and 61 patients (81.3%) were farmers. The mean age and sex were not significantly different between survivors and non-survivors (P = .094 and P = .638, separately). There was also no significant difference with regard to the seasonal incidence (October to December), career, interval from the onset to the patient’s arrival, interval of febrile phase or the frequency of overlapping hypotensive and oliguric phases (P > .05). The survivors tended to have prolonged hospital days compared with the non-survivors (P < .001) (Table 1).

**Table 1:** Demographic, epidemiologic and clinical characteristics in critical patients with HFRS

**Characteristic**	**Survivors (n = 46)**	**Non-survivors (n = 29)**	**P value**^**a**^
**Demographic characteristics**			
Male/Female, n	37/9	22/7	.638
Mean Age, years	48.76 ± 13.59	53.90 ± 11.31	.094
**Epidemiologic characteristics**			
Career (farmer), n (%)	38 (82.6)	23 (79.3)	.721
Incidence (October-December), n (%)	34 (72.3)	16 (55.2)	.125
**Clinical characteristics**			
Interval from onset to the patient’s arrival, days	6.11 ± 6.99	4.93 ± 2.83	.391
Interval of febrile phase, days	4.84 ± 1.68	4.64 ± 1.37	.595
Frequency of overlapping hypotensive and oliguric phases, n (%)	26 (56.5)	22 (75.9)	.089
Hospital days, days	28.35 ± 13.28	7.97 ± 12.33	< .001

^a^Survivors vs. non-survivors.

### Clinical manifestations, supportive treatments, imaging and humoral examinations in the critical patients

Of the clinical symptoms and physical signs, the non-survivors had higher frequencies of dyspnea, agitation, conjunctival hemorrhage and coma (P = .049, P < .001, P = .011, and P < .001, respectively) (Tables 2 and 3). There were no significant differences with respect to the supportive treatments, including the frequencies of renal replacement therapy (RRT) and mechanic ventilation (MV), imaging and humoral examinations, between the survivors and non-survivors (P > .05) (Table 4).

**Table 2:** Symptoms in critical patients with HFRS

**Symptoms**	**Survivors (n = 46)**	**Non-survivors (n = 29)**	**P value**^**a**^
Dyspnea, n (%)	12 (26.1)	14 (48.3)	.049
Fever, n (%)	46 (100)	29 (100)	1.000
Nausea, n (%)	35 (76.1)	21 (72.4)	.722
Vomiting, n (%)	27 (58.7)	17 (58.6)	.995
Anepithymia, n (%)	12 (26.1)	4 (13.8)	.206
Hematemesis, n (%)	5 (10.9)	2 (6.9)	.866
Dark stools, n (%)	7 (15.2)	4 (13.8)	.865
Abdominal distention, n (%)	23 (50)	13 (44.8)	.662
Cough, n (%)	17 (37)	6 (20.7)	.137
Expectoration, n (%)	13 (28.3)	4 (13.8)	.145
Spasm, n (%)	3 (6.5)	3 (10.3)	.875
Agitation, n (%)	16 (34.8)	24 (82.8)	< .001
Fatigue, n (%)	21 (45.7)	10 (34.5)	.339
Lethargy, n (%)	11 (23.9)	10 (34.5)	.323
Hemoptysis, n (%)	2 (4.3)	2 (6.9)	1.000
Blurred vision, n (%)	3 (6.5)	3 (10.3)	.875
Chills, n (%)	10 (21.7)	9 (31)	.367
Diarrhea, n (%)	9 (19.6)	6 (20.7)	.906
Stomachache, n (%)	19 (41.3)	11 (37.9)	.772
Headache, n (%)	22 (47.8)	10 (34.5)	.255
Dizziness, n (%)	13 (28.3)	9 (31)	.797
Lumbago, n (%)	30 (65.2)	14 (48.3)	.147
Chest distress, n (%)	22 (47.8)	13 (44.8)	.800
Short breath, n (%)	22 (47.8)	17 (58.6)	.362

^a^Survivors vs. non-survivors.

**Table 3:** Physical signs in critical patients with HFRS

**Physical signs**	**Survivors (n = 46)**	**Non-survivors (n = 29)**	**P value**^**a**^
Maximum body temperature, °C	39.38 ± 0.60	39.64 ± 0.63	.086
Neck and chest bleeding point, n (%)	23 (50)	16 (55.2)	.662
Conjunctival congestion, n (%)	44 (95.7)	28 (96.6)	1.000
Conjunctival hemorrhage, n (%)	7 (15.2)	12 (41.4)	.011
Soft palate congestion, n (%)	42 (91.3)	26 (89.7)	1.000
Pharyngeal congestion, n (%)	37 (80.4)	21 (72.4)	.419
Eyelid swollen, n (%)	13 (28.3)	10 (34.5)	.569
Edema of lower limbs, n (%)	14 (30.4)	10 (34.5)	.714
Blushing, n (%)	15 (32.6)	6 (20.7)	.263
Positive shifting dullness, n (%)	11 (23.9)	2 (6.9)	.058
Coma, n (%)	6 (13)	22 (75.9)	<.001
Rash, n (%)	4 (8.7)	1 (3.4)	.680
Petechiae, n (%)	26 (56.5)	17 (58.6)	.858

^a^Survivors vs. non-survivors.

**Table 4:** Supportive treatment, imaging and humoral examinations in critical patients with HFRS

	**Survivors (n = 46)**	**Non-survivors (n = 29)**	**P value**^**a**^
**Supportive treatment**			
RRT, n (%)	42 (91.3)	22 (75.9)	.132
CRRT, n (%)	28 (60.9)	22 (75.9)	.180
MV, n (%)	15 (32.6)	7 (24.1)	.433
**Image manifestations**			
Pulmonary effusion, n (%)	31 (62.4)	17 (58.6)	.441
Pleural effusion, n (%)	28 (60.9)	11 (37.9)	.053
Seroperitoneum, n (%)	28 (60.9)	13 (44.8)	.174
T-wave change, n (%)	6 (13)	3 (10.3)	1.000
Premature ventricular contraction, n (%)	5 (10.9)	1 (3.4)	.474
Atrial premature beats, n (%)	3 (6.5)	4 (13.8)	.518
Atrial fibrillation, n (%)	5 (10.9)	3 (10.3)	1.000
Pericardial effusion, n (%)	13 (28.3)	3 (10.3)	.065
Gallbladder swelling, n (%)	10 (21.7)	6 (20.7)	.914
**Humoral examinations**			
Urine protein +++ ~ ++++, n (%)	33 (71.7)	22 (75.9)	.694
Urine occult blood +++ ~ ++++, n (%)	34 (73.9)	17 (58.6)	.167

*Abbreviations:* RRT, renal replacement therapy; CRRT, continuous renal replacement therapy; MV, mechanical ventilation.

^a^Survivors vs. non-survivors.

### HFRS-related complications in the critical patients

Of the HFRS-related complications, the frequencies of cardiac failure, ARDS and encephalopathy in the non-survivors were higher than those in the survivors (P < .001), while the frequency of ARF in the survivors was higher than that of the non-survivors (P = .013) (Table 5). There were no significant differences with respect to the frequencies of pneumonia, arrhythmia, gastrointestinal hemorrhage, hyperglycemia, concurrent bacteremia or secondary hypertension (P > .05).

**Table 5:** HFRS-related complications in critical patients with HFRS

**Complications**	**Survivors (n = 46)**	**Non-survivors (n = 29)**	**P value**^**a**^
ARDS, n (%)	17 (37.6)	25 (86.2)	<.001
Pneumonia, n (%)	41 (89.1)	27 (93.1)	.866
Arrhythmia, n (%)	9 (19.6)	8 (27.6)	.419
Alimentary tract hemorrhage, n (%)	17 (37)	17 (58.6)	.066
Cardiac failure, n (%)	3 (6.5)	11(39.3)	<.001
Hyperglycemia, n (%)	36 (78.3)	25 (86.2)	.390
Encephalopathy, n (%)	11 (23.9)	28 (96.6)	<.001
Concurrent bacteremia, n (%)	13 (28.3)	8 (27.6)	.949
Secondary hypertension, n (%)	26 (56.5)	10 (34.5)	.063
ARF, n (%)	42 (91.3)	20 (69.0)	.013

*Abbreviations:* ARDS, acute respiratory distress syndrome; ARF, acute renal failure.

^a^Survivors vs. non-survivors.

### Laboratory parameters in the critical patients during the acute stage

The levels of white blood cells (WBC), hemoglobin (HGB), alanine aminotransferase (ALT), blood urea nitrogen (BUN), serum creatinine (Scr), glucose, prothrombin time (PT), activated partial thromboplastin time (APTT) and thrombin time (TT) in the critical patients were higher or longer than the reference value (P < .001), while the levels of platelet (PLT) and serum albumin (ALB) were lower than the reference value (P < .001) (Table 6). Compared with the survivors, non-survivors were found to have lower Scr levels (P < .001), prolonged PT (= 0.006) and APTT (= 0.003) and more elevated WBC levels (P = .005) (Table 6).

**Table 6:** Laboratory parameters in critical patients with HFRS during the acute stage

**Variables**	**Survivors (n = 46)**		**Non-survivors (n = 29)**		**P value**^**a**^
	**Mean ± SD**	**n**^**b**^	**Mean ± SD**	**n**^**b**^	
Maximum WBC, 10^9^ cells/L	34.43 ± 16.02^c^	39	47.95 ± 21.78^c^	26	.005
Nadir PLT, 10^9^ cells/L	12.18 ± 11.16^d^	39	14.35 ± 10.28^d^	26	.432
Maximum HGB, g/L	171.00 ± 24.11^c^	39	166.92 ± 26.42^c^	25	.162
Maximum ALT, u/L	170.11 ± 210.92^c^	44	502.91 ± 859.69^c^	29	.213
Nadir serum ALB, g/L	24.53 ± 4.92^d^	44	22.45 ± 5.20^d^	29	.088
Maximum BUN, mmol/L	31.13 ± 8.58^c^	44	26.34 ± 12.77^c^	29	.084
Maximum Scr, μmol/L	778.46 ± 220.86^c^	44	479.66 ± 254.29^c^	29	<.001
Maximum glucose, mmol/L	13.32 ± 6.00^c^	43	15.34 ± 8.22^c^	28	.235
Longest PT, sec	15.20 ± 3.44^c^	45	20.53 ± 9.15^c^	28	.006
Longest APTT, sec	45.96 ± 14.79^c^	45	59.52 ± 19.94^c^	28	.003
Longest TT, sec	23.86 ± 6.24^c^	45	25.32 ± 6.72^c^	28	.282
Minimum Fib, g/L	1.84 ± 0.82^d^	44	1.46 ± 0.84^d^	29	.056

Reference values: WBC (3.2-9.7) × 10^9^/L; PLT (100–300) × 10^9^/L; HGB (120–160) g/L (male), (110–150) g/L (female); ALT (4–44) U/L; serum ALB (35–55) g/L; serum BUN (2.86-8.20) mmol/L; serum Cr (53–97) μmol/L (male), (35–71) μmol/L (female); glucose (3.89-6.11) mmol/L; PT (8.8-13.8) sec; APTT (25.1-36.5) sec; TT (10.3-16.6) sec, Fib (2.38-4.98) g/L.

*Abbreviations:* WBC, white blood cells; PLT, platelet; HGB, hemoglobin; ALT, alanine aminotransferase; ALB, albumin; BUN, blood urea nitrogen; Scr, serum creatinine; PT, prothrombin time; APTT, activated partial thromboplastin time; TT, thrombin time; Fib, fibrinogen.

^a^Survivors vs. non-survivors.

^b^Some critical patients had passed through the acute stage on admission, and therefore, no laboratory data were available.

^c^Higher or longer than the reference value, P < .001.

^d^Lower than the reference value, P < .001.

### Spearman correlation and logistic regression analysis

Of the clinical manifestations, agitation, conjunctival hemorrhage, and coma were negatively correlated with the survival outcome (P < .05) (Table 7). Of the HFRS-related complications, cardiac failure, ARDS and encephalopathy were negatively correlated with the survival outcome, while ARF was positively correlated with the survival outcome (P < .05) (Table 7). Of the laboratory parameters, WBC, PT and APTT were negatively correlated with the survival outcome, while Scr was positively correlated with the survival outcome (P < .05) (Table 7).

**Table 7:** Spearman correlation analysis in critical patients with HFRS

	**Survival**			**Survival**	
**Parameters**	**r**	**P value**	**Parameters**	**r**	**P value**
Agitation	-.468	< .001	Encephalopathy	-.743	< .001
Conjunctival hemorrhage	-.293	.011	ARF	.287	.012
Coma	-.632	< .001	WBC	-.330	.006
Dyspnea	-.227	.051	Scr	.550	< .001
ARDS	-.512	< .001	PT	-.370	.001
Cardiac failure	-.393	< .001	APTT	-.362	.002

*Abbreviations:* r, correlation coefficient; ARDS, acute respiratory distress syndrome; ARF, acute renal failure; WBC, white blood cells; Scr, serum creatinine; PT, prothrombin time; APTT, activated partial thromboplastin time.

To explore risk factors for prognosis, binary logistic regression analysis was used to analyze the clinical symptoms, signs and complications that were correlated with the survivor outcome. The prognosis of the critical patients was stratified with death defined as “1” and survival defined as “0”. The clinical parameters were further stratified and ranked according to their occurrence during the clinical course. Finally, ARDS, conjunctival hemorrhage and coma were identified as risk factors for prognosis, with odds ratios (ORs) of 14.333, 17.640 and 25.716 respectively (P < 0.05). Regression coefficients of the three factors (Table 8) were used to calculate a logit of death as follows:

$$\begin{array}{l} \mathrm{The}\;\mathrm{logarithm}\;\mathrm{of}\;\mathrm{odds}\;\mathrm{of}\;\mathrm{death}\\ \;=‒5.295+2.663\;\mathrm{ARDS}\\ \;+2.870\;\mathrm{Conjunctival}\;\mathrm{hemorrhage}\\ \;+3.247\;\mathrm{Coma} \end{array}$$

**Table 8:** Independent risk factors for death in critical patients with HFRS

**Parameters**	**B**	**SE**	**Wald**	**df**	**P**	**OR**	**95% CI for OR**	
							**Lower**	**Upper**
ARDS	2.663	1.215	4.799	1	.028	14.333	1.324	155.222
Conjunctival hemorrhage	2.870	1.064	7.275	1	.007	17.640	2.191	141.989
Coma	3.247	.927	12.257	1	.000	25.716	4.176	158.372
Constant	-5.295	1.641	10.409	1	.001	.005		

Forward: conditional; Omnibus Test of Coefficients: Chi-square 53.226, P < .001.

*Abbreviations:* B, independent variable coefficient; SE, standard error; CI, confidence interval; df, degrees of freedom; OR, odds ratio.

### ROC and Kaplan-Meier survival analysis

**Figure 1:**
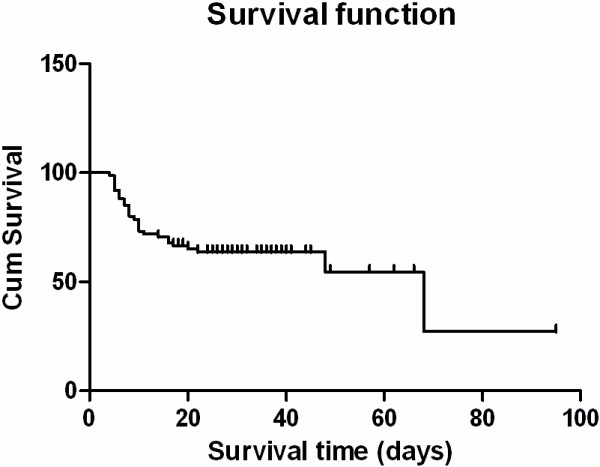
**The Kaplan-Meier survival analysis is based on the total survival time in the period from the onset of illness to discharge or death in the critical patients with HFRS.** The event of interest was defined as patient death during hospitalization and patient discharge as censored. Of the 75 critical patients, the cumulative proportion surviving of 85.3% at the first week interval, 70.7% at the second week interval, 65.1% at the third week interval and 63.7% at the fourth week interval.

Kaplan-Meier analysis was applied to determine the cumulative proportion surviving and 28-day mortality rate in the critical patients. The analysis revealed that the cumulative proportion surviving was 70.7% at the second week interval, with a 28-day mortality rate of 36.3% (Figure 1).

**Figure 2:**
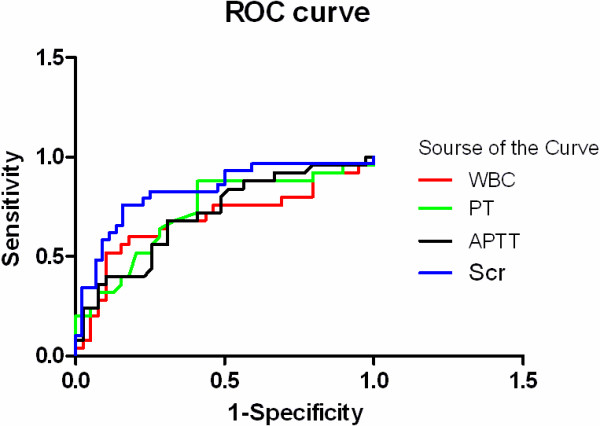
**ROC analysis of WBC, Scr, PT and APTT revealed that the four parameters reached statistical significance for predicting prognosis (P < .05).** Abbreviations: ROC, receiver operating characteristic; WBC, white blood cells; PT, prothrombin time; APTT, activated partial thromboplastin time; Scr, creatinine.

To explore the predicting value for prognosis on the laboratory parameters, ROC and AUC were analyzed. The analysis revealed that the WBC and Scr levels and the PT and APTT times were statistically significant for predicting prognosis (P < .05) (Figure 2, Table 9).

**Table 9:** Predictive values for prognosis on laboratory parameters in critical patients with HFRS

**Parameters**	**AUC**	**P value**^**a**^	**Cut-off value**	**Sensitivity**^**b**^	**Specificity**^**b**^	**95% Cl for AUC**^**b**^	
						**Lower**	**Upper**
WBC	0.688	0.012	46.05	60.0	82.1	54.4	83.2
Scr^c^	0.828	0.001	586.7	76.0	84.1	72.7	93.0
PT	0.724	0.003	15.60	88.0	59.0	59.3	85.6
APTT	0.710	0.005	52.10	69.0	69.2	58.0	84.0

*Abbreviations:* AUC, area under the curve; CI, confidence interval.

^a^P value for calculated AUC in predicting death.

^b^Sensitivity, specificity and 95% CI are all presented as percentages.

^c^Test direction: lower test result indicates a more positive test.

## Discussion

To our knowledge, this is the first clinical study on the clinical characteristics and outcomes in critical patients with HFRS, with the largest sample enrolled in China. In the 75 critical patients enrolled, 29 cases died, with a hospital fatality of 38.7%. The Kaplan-Meier analysis revealed that the cumulative proportion surviving was 70.7% at the second week interval, with a 28-day mortality rate of 36.3% (Figure 1). This observation indicated that the fatality of critical patients with HFRS was high and was similar to the mortality rate of severe septic shock patients in the intensive care unit (ICU) [18-20]. In this study, a majority of patients enrolled were male and farmers, with high incidence and obvious seasonal distribution (Table 1). These data are consistent with the biological and epidemical characteristics of the primary natural host (*Apodemus agrarius*) of HTNV and the susceptibility of the patients working in certain surroundings that are the natural habitat of rodents in Xi'an city [6,7,21]. Until now, the hantavirus strains from host rodents and patients in Xi’an only belong to the HTNV, and SEOV strains have not been found [6].

In this study, the survivors tended to have prolonged hospital stays (Table 1), which were consistent with the clinical course of HFRS. Generally, the hypotensive phase of HFRS (e.g., low blood pressure and circulation collapse) usually occurs between day 3 and day 7 of the clinical course, and some critical patients even exhibit overlapping hypotensive and oliguric phases combined with various fatal complications. During this period, timely and systematically supportive treatment, including mechanical ventilation combined with continuous blood purification (CBP), vasoactive drugs and nutritional supplement, are necessary to ensure survival. However, some critical patients still died of refractory shock, ARDS, multiorgan bleeding, disseminated intravascular coagulation (DIC) or multiple organ dysfunction syndrome (MODS). Patients who have passed through the critical phase may manifest obvious oliguria with AKI. The durations of the oliguric phase in patients with HFRS are obviously different based upon the degree of AKI [16] and the influence on applications of RRT including continuous renal replacement therapy (CRRT) and intermittent hemodialysis (IHD) [22,23]; only a minority of critical patients die during this phase. Furthermore, it is an unfortunate truth that in addition to the critical condition of the disease on admission, the patient’s economic status may also influence the prognosis directly or indirectly.

The present study demonstrated that the non-survivors had higher frequencies of dyspnea, agitation, conjunctival hemorrhage, coma, cardiac failure, ARDS and encephalopathy compared to the survivors (Tables 2, 3, 4 and 5). The agitation, conjunctival hemorrhage, coma, cardiac failure, ARDS and encephalopathy were all negatively correlated with the survival outcome (Table 7). Although there was no significant relationship, the difference was obvious in pleural effusion and pericardial effusion found in the survivors vs. non-survivors (Table 4). According to the pathophysiologic characteristics of HFRS, the pleural and pericardial effusion can reflect the degree of body edema and may increase the risk of the occurrence of pulmonary infection, ARDS and heart failure. All of these findings highlight that it would be dangerous to ignore the critical condition of the disease and the increased probability of death when patients exhibit clinical manifestations of ARDS and encephalopathy, and conjunctival hemorrhage can be considered to be a local manifestation of severe organ bleeding with poor outcome. Furthermore, ARDS, conjunctival hemorrhage and coma were identified as risk factors for prognosis by binary logistic regression analysis (Table 8), which underscores the importance of clinicians’ alertness to the occurrence of potentially fatal complications. Additionally, although cardiac failure was not a risk factor for death in the present report, it deserves further study in larger samples of critical patients, especially the cases of deaths.

Kidney is a major organ that is damaged during HFRS, with the most prominent pathological presentation being acute tubulointerstitial nephritis following the infiltration of inflammatory cells [24]. In this study, a majority of the patients had humoral abnormalities with obvious urine protein and urine occult blood (Table 4), which may reflect the degree of severity of AKI. In this study, all the critical patients had different stages of AKI, and 62 cases (82.7%) exhibited ARF, the third stage of AKI [16] (Table 5). Interestingly, we observed that the frequency of ARF in the survivors was obviously higher than that of the non-survivors (Table 5). ARF and Scr were positively correlated with the survival outcome (Table 7); non-survivors were also found to have lower Scr levels (Table 6).These results can be explained by the fact that some critical patients died during the overlapping phase because of other fatal complications when ARF was not obviously detected. According to the data, we can conclude that the stage of AKI and the duration of the oliguric phase during the acute stage are not a directly influential factor for prognosis because of the application of RRT.

This study observed that the levels of WBC, HGB, ALT, BUN, Scr, glucose, PT, APTT and TT in the critical patients were higher or longer than the reference value, while the levels of PLT and ALB were lower than the reference value (Table 6), which indicated that critical patients with HFRS usually had obvious hepatic and kidney injury, and stress hyperglycemia and hypermetabolism were also common during the clinical course. According to the pathophysiologic mechanism of HFRS, it has been widely accepted that severe plasma leakage, massive bleeding and/or profound shock may lead to tissue hypoperfusion, potentially rendering AKI and hepatic injury [25]; the high level of HGB was closely related to the degree of pachyhemia; the low level of PLT was related to the decreased platelet production or increased platelet destruction [26-28]; the low level of ALB correlates with degree of the loss of vascular integrity and enhanced vascular permeability, which also usually appears in patients with dengue hemorrhagic fever (DHF) [3,29]. Compared to the survivors, the non-survivors were found to be more critical, with a higher degree of inflammatory reaction and coagulation abnormality, which is in line with the previous reports on patients with DHF [30-32]. Furthermore, although the AUCs were not higher than 0.900 and would be influenced by small samples or treatments, ROC analysis still revealed that the WBC levels, PT and APTT times were statistically significant for predicting death (Table 9, Figure 2). In total, the laboratory results may reflect the severity of the disease to a degree, and clinicians should be alert to their dynamic changes during the acute stage, ensure close monitoring and thereby initiate a timely management as necessary.

As a retrospective study, some limitations must be addressed. First, this study was conducted at the largest center for infectious diseases in the northwest region of China. Most patients with severe clinical conditions were sent to our medical center, but it is likely that outside of our center, there were still a significant number of critical HFRS patients who were admitted to local hospitals. Therefore, the demographic, epidemiological characteristics and mortality rate in critical patients may have been biased. Second, the clinical manifestations may be biased by clinicians based on his or her personal recognition of the clinical severity. In this study, we were unable to calculate the acute physiology, age, chronic health evaluation (APACHE II) score, sepsis related organ failure assessment (SOFA) and simplified acute physiology score (SAPS II) of the critical patients on admission because of the loss of detailed clinical data on central nervous system dysfunction. These data may be very important for predicting the prognosis. Third, the relatively small number of cases made the statistical power quite small, especially for multivariate logistic regression analysis used for identifying risk factors for death in the critical patients. Finally, the clinical outcomes and classifications of HFRS patients might be biased by the lack of a more standardized protocol for the management of patients with HFRS.

## Conclusion

The high fatality in critical patients with HFRS underscores the importance of clinicians’ alertness to the occurrence of potentially fatal complications and changes in biochemical status to ensure that timely and systematically supportive treatment can be initiated when necessary.

## Abbreviations

HFRS: Hemorrhagic fever with renal syndrome; ARDS: Acute respiratory distress syndrome; Scr: Serum creatinine; PT: Prothrombin time; APTT: Activated partial thromboplastin time; WBC: White blood cells; HGB: Hemoglobin; ALT: Alanine aminotransferase; BUN: Blood urea nitrogen; TT: Thrombin time; PLT: Platelet; ALB: Albumin; AKI: Acute kidney injury; HTNV: Hantaan virus; ELISA: Enzyme-linked immunosorbent assay; CT: Computed tomography; ARF: Acute kidney failure; SBP: Systolic blood pressure; ECG: Electrocardiography; RRT: Renal replacement therapy; MV: Mechanic ventilation; ICU: Intensive care unit; CBP: Continuous blood purification; DIC: Disseminated intravascular coagulation; MODS: Multiple organ dysfunction syndrome; CRRT: Continuous renal replacement therapy; IHD: Intermittent hemodialysis; DHF: Dengue hemorrhagic fever.

## Competing interests

The authors declare that they have no competing interests.

## Authors’ contributions

HD, PZW and XFB conceived the study, and participated in its design and coordination. HD, JL, JNW and HTY reviewed and collected the data. HD, LB, YZ, WJ and HL analyzed and interpreted the data. HD drafted the manuscript. PZW and XFB reviewed the manuscript. All authors approved the final manuscript.

## Pre-publication history

The pre-publication history for this paper can be accessed here:

http://www.biomedcentral.com/1471-2334/14/191/prepub
